# Shelf help: what powers medical student subject exam performance in the United States

**DOI:** 10.1080/10872981.2026.2708427

**Published:** 2026-08-01

**Authors:** Corine Astroth, Tyler Lunow-Luke, Maren Smith, Josephine Chang, Areg Grigorian, Michael P. O’Leary

**Affiliations:** a Department of Surgery, School of Medicine, University of California Irvine, Orange, California, United States of America

**Keywords:** Shelf exam, subject exam, NBME, USMLE, medical education

## Abstract

**Background:**

Following the change in 2022 of a scored United States Medical Licensing Examination (USMLE) Step 1 to a binary pass-fail grading system, greater emphasis has been placed on the Step 2 Clinical Knowledge (Step 2 CK) exam. Shelf or subject exams offered by the National Board of Medical Examiners (NBME) help prepare students for Step 2CK and therefore deserve the attention of clerkship directors. This paper reviews the literature pertaining to the factors that positively and negatively impact medical students' performance on the shelf exams, including but not limited to performance in the pre-clinical years, clinical exposures, peer learning, active or team-based learning, length of clerkships, and order and number of previous shelf exams.

**Methods:**

The PubMed database was searched to identify studies describing factors influencing performance on shelf exam scores during medical school and strategies for improving shelf scores. Studies were identified through iterative searches using multiple keyword combinations related to NBME shelf examinations, NBME subject examinations, clerkship performance, academic achievement, and educational interventions. Inclusion criteria consisted of English-language studies published between 2005 and 2025 involving students at U.S. allopathic or osteopathic medical schools. The included studies were reviewed for relevance and methodological rigour, including consideration of study design, sample size, statistical analyses, and reported outcomes.

**Results:**

A robust pre-clinical education remains vital for success on NBME shelf examinations. Exam scores are also influenced by the number and order of previous exams. Increased clinical exposure, active learning, team-based approaches, and peer review sessions have not consistently shown improvements in performance.

**Conclusions:**

This review underscores the importance of a foundational understanding of the basic sciences for student success on exams, while acknowledging that clerkship directors must balance their obligation to adequately prepare students for standardized testing with their mission to craft compassionate and competent future clinicians.

## Introduction

Throughout the clerkship years of medical school, students are required to take national standardized exams known as subject or shelf exams administered by the National Board of Medical Examiners (NBME). These exams, referred to in this paper as shelf exams (Table 4), are administered in the core clerkship rotations, including Medicine, Surgery, Family Medicine, Clinical Neurology, Psychiatry, Pediatrics, Obstetrics and Gynecology, and Ambulatory Care. At many schools, these scores play a role in determining students’ final clerkship grades or pass/fail status and therefore influence the transcripts that will eventually be submitted to the Electronic Residency Application Service (ERAS). Additionally, the shelf exams are considered essential in preparation for the Step 2 Clinical Knowledge exam (Step 2 CK), as scores on the exams are positively correlated with eventual Step 2 CK scores [1]. In fact, a 5-point increase in the medicinal shelf exam score was associated with a 4.65-point increase in Step 2 CK performance [2]. These findings warrant additional attention, given that Step 1 is now a binary pass or fail, and many schools have moved away from grades in the pre-clerkship and clerkship curriculums. Therefore, Step 2 CK is one of the few remaining objective measures to evaluate students applying for residency and continues to be one of the main factors associated with a medical student’s successful application to residency [3–5]. As such, medical school administrators and clerkship directors have a responsibility to ensure their students are well prepared for the shelf exams. Here, we review the current literature relating to factors that may contribute to higher shelf exam scores and suggest how these insights could be applied to medical curriculum.

## Methods

The PubMed database was searched to identify studies describing factors influencing performance on shelf exam scores during medical school and strategies for improving shelf scores. Given the heterogeneity within the research on this topic with respect to clerkship specialties, institutional characteristics, education interventions, outcome measures, and study designs, a narrative review structure was selected. Studies were identified through iterative searches using multiple keyword combinations related to NBME shelf examinations, NBME subject examinations, clerkship performance, academic achievement, and educational interventions. Inclusion criteria consisted of English-language studies published between 2005 and 2025 involving students at U.S. allopathic or osteopathic medical schools. Included studies were reviewed for relevance and methodological rigour, including consideration of study design, sample size, statistical analyses, and reported outcomes.

## Results and discussion

### Correlation of pre-clinical performance

In recent years, multiple well-established medical schools have moved away from the traditional two-year preclinical and two-year clinical model of medical education to curriculums that increase early clinical experiences. For example, Duke medical students complete their preclinical work in 11 months, while University of California San Francisco (UCSF) and Harvard medical students take 18 months. These changes are not isolated events. A 2025 JAMA study found that of 157 medical schools surveyed, 69.5% had shortened their pre-clinical curriculum to 1.5 years or less [6]. Such changes have been found to be associated with lower shelf exam scores [7,8], and there is a growing body of evidence to suggest that the foundational medical knowledge, gained during the preclinical years or otherwise, may be essential for future success on shelf exams.

Step 1 has classically been used as the final exam before students proceed to their clerkships, as it covers much of the basic science knowledge gathered in the pre-clinical years. Although this timeline is now changing at many institutions, Step 1 scores can still be considered a benchmark of student understanding of foundational science. Performance on Step 1 has been found to be a powerful predictor of performance on multiple shelf exams [1,9,10], and moving Step 1 to after clerkships was associated with statistically significant lower performance on four of the shelf exams [11]. Step 1 scores also significantly predicted between 23% and 44% of the variance in shelf exam scores for all clerkships [12]. Additionally, it has been reported that the single strongest predictor of failure on the surgery shelf exam was performance on the preclinical pathology shelf exam – the content of which focuses on the general principles of foundational science, including the biology of cells and tissue response to disease above all other topics [13]. These results are mirrored by a similar study that showed pathology course scores were the strongest predictor of performance on *all* future clinical shelf exams and Step exams [14]. The above findings boast relatively large sample sizes across multiple institutions, as shown in Table 1, and raise concerns for the modern medical educator given the ongoing changes to the pre-clinical timeline. The findings for pathology in particular offer a window into the current challenges educators face. With the decreased time spent in the pre-clinical years, schools have moved away from offering a stand-alone pathology course despite documented challenges of integrating pathology education into clinical settings [15]. Notably, these changes have occurred alongside the growing use of third-party resources, such as Pathoma, without any literature to demonstrate their equivalence or superiority [16,17].

**Table 1.** Performance on exams testing foundational knowledge is associated with better performance on shelf exams.

Title	Author and Year	Sample size	Outcome	*p*-value	Takeaway for curriculum development
Correlation of National Board of Medical Examiners scores with United States Medical Licensing Examination Step 1 and Step 2 scores	Zahn et al. [1]	507	Step 1 and 2 exam scores have moderate to large positive correlations with all subject exam scores	<0.001	
Which Internal Medicine Clerkship Characteristics Are Associated With Students’ Performance on the NBME Medicine Subject Exam? A Multi-Institutional Analysis	Fitz et al. [9]	24,542	Step 1 performance is the most powerful predictor of Medicine exam performance	<0.001	
Factors associated with performance in an internal medicine clerkship	Colbert et al. [10]	341	Step 1 performance accounted for the most unique variance in subject exam score	<0.001	
Does Delaying the United States Licensing Examination Step 1 to After Clerkships Affect Student Performance on Clerkship Subject Examinations?	Jurich et al. [11]	23,677	Delaying Step 1 to after clerkships results in lower scores for Medicine*, Pediatrics*, Neurology*, and Surgery** but is not associated with changes for OBGYN*** or Psychiatry****	<0.001*, <0.002**, 0.99***, 0.15****	*An understanding of the basic sciences and disease pathology is essential for success on NBME subject exams.*
Are Scores From NBME Subject Examinations Valid Measures of Knowledge Acquired During Clinical Clerkships?	Ryan et al. [12]	432	Step 1 scores predicted between 23%–44% of variance in subject exam scores	<0.001	
Preclinical predictors of surgery NBME exam performance	Kozar et al. [13]	194	The single strongest predictor of failure on the surgery NBME found to be performance on the pathology shelf exam	<0.0001	
Relationships between preclinical course grades and standardized exam performance	Hu et al. [14]	795	Pathology course score found to be the strongest predictor of performance on all subject exams and Step exams	<0.001	

### Correlation of clinical performance

Basic science knowledge even appears to play a larger role than clinical performance when it comes to shelf exam scores, as the clinical evaluations of medical students were only negligibly correlated with scores on the surgery shelf [18] and not at all correlated with scores on the psychiatry shelf [19]. It has been suggested that clinical exposure is highly beneficial for shelf exam preparation by exposing students to a more diverse pathology during their rotations; however, this has not been found to be the case. Students placed in various locations for psychiatry clerkships, including but not limited to the VA, regional tertiary hospitals, or acute care private care psychiatric services, did not have significant differences in their shelf exam performance [20]. Similarly, another study completed on students who were either exposed to general surgery or strictly subspecialty surgery during their surgery clerkship noted no difference in their shelf performance [21]. The fact that the different levels of clinical exposure and thus the subsequent diversity of pathologies observed played no role in shelf scores suggests that knowledge gathered from the clinical setting may not be sufficient for success on the shelf examinations.

Despite these findings, other studies in circulation appear to counter the above points. The Objective Structured Clinical Examination or OSCE, which is often incorporated into final clerkship grades alongside evaluations and shelf scores, has proven to be significantly positively associated with shelf exam scores [22]. After Step 1 scores, OSCE scores were found to account for the most unique variance in the internal medicine shelf exam when compared to the MCAT, second-year GPA, and clinical evaluations [10]. It may be worthwhile to investigate why rotations with greater clinical exposure are not associated with increased shelf exam scores, while the OSCE, a clinical exam, is. One hypothesis is that although the OSCE is a clinical exam, it is also standardized within an institution. Given this, many of the pertinent questions a student must ask to gain full points are still based on the classic presentation of disease, which often ultimately emphasizes the basic disease pathology tested by NBME.

Other notable findings in the literature include an association between the number of ‘fresh’ patients students cared for during their rotation versus hand-off patients that they inherited from other members of the care team. The number of fresh patients was associated with an exam percentile ranking increase of 0.2 points [23]. Again, it would be worth investigating why exposure to disease pathology was not associated with increased scores, while the number of ‘fresh’ patients was. While it is possible that site assignment does not serve as a strong proxy for diversity of diseases encountered, perhaps diversity is ultimately less important than a thorough understanding of the management that is encouraged by a ‘fresh’ patient encounter. Educators should be made aware that the age-old adage ‘quality over quantity’ likely still holds true for medical education today.

Finally, there is evidence of at least one intervention that encouraged clinical reasoning that was also associated with higher shelf scores. A family medicine clerkship assigned approximately one-hour-long web-based cases that were meant to improve diagnostic and clinical reasoning skills [24]. Students who completed these cases did better on the shelf even when controlling for prior academic performance and clerkship timing. This emphasizes that while the importance of basic science and disease pathology should not be underestimated, it is also possible that successful curriculum changes simultaneously focus on clinical skills. This study, as shown in Table 2, has a relatively smaller sample size and was conducted at a single institution. Its success, to our knowledge, has not been replicated elsewhere, underlining the ongoing need for research into why and how only some clinical interventions are successful.

**Table 2.** Greater involvement in clinical activities and skill building does not uniformly lead to higher shelf exam scores.

Title	Author and Year	Sample Size	Outcome	*p*-value	Takeaway for Curriculum Development
Surgical clerkship: Do examination scores correlate with clinical performance?	Saberi et al. [18]	625	Surgical clerkship clinical performance was negligibly correlated with performance on surgery NBME exam	<0.001	
Grading medical students in a psychiatry clerkship: correlation with the NBME subject examination scores and its implications	Ramchandani [19]	141	Grades in clinical reasoning, data-synthesis, and history taking did not predict shelf exam score	NA	
Choice of four primary clinical placements in a one-month clerkship: who performs the best on the shelf exam, and what does it mean?	Rosenthal et al. [20]	118	No difference in shelf exam scores based on clinical placement location	<0.891	
Impact of general surgery rotation exposure on surgery clerkship performance	Hawley et al. [21]	365	Performance on NBME was comparable for students exposed to general surgery versus subspecialty surgery	0.51	*Clinical exposures and skills, while important for crafting competent future physicians, are not consistently associated with improved NBME subject exam scores. Despite this, the OSCE, a clinical exam, has been positively associated with performance on subject exams.*
Does a surgical observed structured clinical exam (OSCE) predict clerkship grade, shelf exam scores, and preceptor clinical evaluation?	Brallier et al. [22]	392	The OSCE is positively associated with shelf exam performance with a linear trend when moving from fail to pass to high pass to honours	<0.001	
Association between hand-off patients and exam performance in the medicine clerkship	Lang et al. [23]	89	Number of full evaluations completed on fresh patients positively associated with subject exam percentile	0.048	
Relating student performance on a family medicine clerkship	Shokar et al. [24]	196	Students who completed family medicine web-based cases scored higher on the NBME family medicine exam	<0.000	

### Attempted interventions

The diversity of topics in the above discussion also highlights that there is no standard national curriculum for shelf exams, only a content outline provided by the NBME. This has likely contributed to the recent interest in the literature for educational interventions that increase student shelf exam scores.

The importance of a strong understanding of foundational science carries over into published work on attempted curriculum interventions and their relative successes. Interventions that emphasized basic science over clinical reasoning fared better when it came to shelf exams, but not always in terms of student satisfaction. An intervention at UCSF Medical School led by fourth-year medical students that included sessions on test-taking strategies, script development, and radiology review was associated with statistically significantly *lower* shelf exam scores when compared to students who did not participate in the sessions [25]. However, students reported wanting more of these sessions incorporated into future clerkships. Conversely, in another UCSF intervention involving didactic sessions designed to solidify foundational clinical science knowledge, students who participated in the course scored similarly to the national average on NBME surgical shelf self-assessments, while the students who did not participate scored lower than the national average [26]. Despite this, didactic sessions were removed from future clerkships based on student feedback requesting additional time for clinical experiences. These studies were limited by being smaller studies completed at one institution. However, other studies have shown that review sessions focusing on disease pathology produced statistically significant improvements in shelf exam scores [27,28] while interventions aimed at critical thinking and clinical skills led to worse shelf performance [29]. This underlines the point that course directors concerned with student success on exams should focus education around basic disease comprehension and continues to raise questions about the shortened pre-clinical timeline. It is possible that the failed clinical reasoning interventions are due to the shelf exams not properly capturing clinical knowledge, and it is also possible that the development of such skills relies first on the core knowledge of disease pathology. Students cannot be expected to move to critical reasoning without the proper foundation. The field of medical education needs to determine which of these possibilities, if either, offers the true explanation for the observed discrepancies in order for real progress to be made.

We have not yet seen a movement to answer such questions, but we have noted other recent trends in education. Across the board, there has been a push towards active learning, often taking the form of flipped classroom and team-based learning models [30,31]. Given their rising popularity and purported success, it is no surprise that these techniques have been implemented to help improve shelf scores. Unfortunately, the results of such interventions have been suboptimal.

At one institution that looked at third year medical students before and after team-based learning was added to the surgery clerkship, students’ NBME shelf performance was significantly lower after intervention although authors note that scores were also lower nationally [32]. Despite the lack of improvement, students enjoyed the sessions and were more likely to rate the clerkship as good and/or excellent after the curriculum changes. Similarly, another institution that added a flipped classroom element to its surgery clerkship found no significant difference [33]. Yet again, researchers observed that the majority of students preferred the new teaching methods, a finding replicated at another institution with flipped classroom sessions in the surgery clerkship [34].

Similar trends have been noted when looking at active learning in psychiatry. Students who spent more time in active learning environments, defined as team-based learning, small group activities or clinical simulations, did not fare significantly better regarding their shelf scores [35]. And in a similar study, despite there not being significantly different scores between students taught via active learning or traditional lectures, the active learning students had greater satisfaction with their courses [36]. These findings are consistent with findings in medical education in general, with a 2016 review of flipped classroom models in medical education that evaluated eleven studies and found that while four studies reported improvements, four showed no difference, and the remaining three had mixed results, leading the authors to conclude the findings were equivocal [37].

Despite the lack of evidence to support various active learning modalities when it comes to increasing shelf scores (Table 3), it is clear that students overall enjoy learning more when changes are made that engage them in their education. This is not a negligible fact for clerkship directors. Since active learning does not appear to decrease scores, increasing such opportunities could still be justified on the grounds of student happiness, satisfaction, or wellness at large (Table 4).

**Table 3.** Interventions emphasizing foundational concepts and the timing of Shelf exams influence scores, while the promotion of clinical reasoning skills and critical analysis does not.

Title	Author and Year	Sample Size	Outcome	*p*-value	Takeaway for Curriculum Development
Near-Peer Learning During the Surgical Clerkship: A Way to Facilitate Learning After a 15-Month Preclinical Curriculum	Hernandez et al. [25]	104	Average surgical shelf score of individuals who attended near pear learning sessions was lower than students who did not participate	0.04	*Interventions emphasizing foundational concepts fare better than those focused on clinical reasoning skills or critical analysis, and while students appear to enjoy active and team-based learning, there is not an associated improvement in scores. Clerkship directors should also be aware that shelf exams are influenced by where they fall in a student's personal schedule.*
Where Do We Go From Here? Assessing Medical Students’ Surgery Clerkship Preparedness During COVID-19	Nnamani et al. [26]	82	Students who completed EMLR had an average NBME surgical self-assessment score of 72 compared to score of 66 for students in traditional clerkship and 69 for students in longitudinal clerkship	Not reported	
Psychiatry resident-led tutorials increase medical student knowledge and improve national board medical examiners shelf exam scores	McKean & Palmer [27]	188	Medical students NBME shelf exam score during the year tutorials were added was higher than the four previous years	0.002	
Impact of a structured review session on medical student psychiatry subject exam performance	Siddiqi et al. [28]	159	Mean score with review session was 87.8 compared to previous 85.3 and the mean for the lowest two scores in each student group improved from 74.1 to 78.7	0.03; 0.0007	
Curricular Innovation in the Surgery Clerkship: Can Assessment Methods Influence Development of Critical Thinking and Clinical Skills?	McClintic et al. [29]	141	Shelf exam mean in intervention group was found to be 67.5 versus 75.5 in those not exposed to critical thinking and clinical skills curriculum	<0.0001	
Team-Based Learning in the Surgery Clerkship: Impact on Student Examination Score, Evaluations, and Perceptions	Kaminski et al. [32]	407	Mean NBME surgery scores decreased following TBL sessions (77.10 vs. 74.65) but notably were also lower nationally (75.20 vs. 73.10).	0.039; <0.001	
Implementation of a flipped classroom approach to promote active learning in the third-year surgery clerkship	Lewis et al. [33]	200	There was no difference in Step 1 scores between group exposed to flipped classroom model and those on the traditional model; likewise, shelf exam scores were not different between groups	0.13; 0.50	
Student perceptions of a simulation-based flipped classroom for the surgery clerkship: A mixed-methods study	Liebert et al. [34]	76–77	Students had strong positive feedback for the flipped classroom approach	NA	
Impact of Varying Active Learning Time of Student Performance on a Standardized Exam in the Psychiatry Clerkship	Crisafio & Cho [35]	518	Psychiatry NBME subject exam scores were not improved by increasing the amount of active learning; however, taking the clerkship later in the year and after internal medicine was found improve performance	0.402; <0.0001; <0.0001	
Incorporating Active Learning Into a Psychiatry Clerkship: Does It Make a Difference?	Morreale et al. [36]	210	Satisfaction was increased in the active learning group, but psychiatry NBME scores were not improved	0.013; >0.05	
Shorter psychiatry clerkship length is associated with lower performance	Bostwick & Alexander [38]	516	Scores on the psychiatry NBME were reduced in those who completed the clerkship in 3-weeks or 4-weeks compared to 6-weeks	0.03; 0.01	
Surgical Education and the Longitudinal Model at the Columbia-Bassett Program	Charak et al. [39]	148	The longitudinal surgical curriculum cohort outperformed the standard curriculum cohort on NBME	0.001	
Does Reducing Clerkship Lengths by 25% Affect Medical Student Performance and Perceptions?	Monrad et al. [40]	515	Shortening clerkships by 25% did not result in significant differences in performance on NBME subject exams between immediately preceding clerkship and traditional cohort	>0.002 (corrected significance value)	
Time Does Matter: Reduced Internal Medicine Clerkship Clinical Experiences Due to COVID-19	Coronel-Couto et al. [41]	235	Students with shortened IM clerkship clinical care time had no statistical difference in NBME subject exam performance	<0.4951	
Neurology Clerkship: Predictors of Objective Structured Clinical Examination and Shelf Performance	Sampat et al. [42]	439	Only the number of completed core clerkships had an impact on the NBME neurology exam scores	0.007	
Timing Bias in the Psychiatry Subject Examination of the National Board of Medical Examiners	Manley & Heiss [43]	635	Students who took the NBME Psychiatry exam at the end of their clerkship sequence scored higher than those who took it at the beginning	NA	
Measuring what matters: identifying assessments that reflect learning on the core surgical clerkship	Mikulski et al. [44]	192	Surgery shelf was influenced by timing before or after Internal Medicine. Students did significantly better on their surgery shelf if taken after IM	<0.001	
Factors Associated With Surgery Clerkship Performance and Subsequent USMLE Step Scores	Dong et al. [45]	687	The students who finished the internal medicine clerkship before the surgery clerkship achieved significantly higher surgery NBME subject exam scores	<0.01	

**Table 4.** Terminology key.

Term	Explanation
National Board of Medical Examiners (NBME)	Non-profit, independent organization that develops and administers assessments for medical students, residents, and physicians.
Subject exam OR shelf exam OR NBME exam	Standardized multiple-choice exams typically 165 min in length with 110 specialty-specific questions created by NBME to assess medical student knowledge.
Electronic Residency Application Service (ERAS)	Online medical residency application system developed by the Association of American Medical Colleges (AAMC).
United States Medical Licensing Examination (USMLE) Step 1	First of three examinations required for medical licensure in the United States, co-sponsored by both the NBME and the Federation of State Medical Boards (FSMB).
USMLE Step 2 Clinical Knowledge (Step 2 CK)	Second of three examinations required for medical licensure in the United States, co-sponsored by both the NBME and the FSMB.
Third-party resources	Study materials and learning tools outside the official medical school curriculum. Includes commercially produced resources such as question banks, review books, flashcard systems, and podcasts.
Pathoma	Popular third-party resource created by Dr. Husain A. Sattar that is specific to pathology and includes a textbook with accompanying video lectures.
Objective Structured Clinical Examination (OSCE)	Performance-based assessment standardized within one medical institution where students rotate through a series of timed stations where they interact with standardized patients and perform clinical tasks.
Peer-learning	Educational approach that emphasizes students learning from and/or with each other often utilizing collaborative activities.
Active-learning	Instructional approach that requires learners to engage directly with material often through activities that require analysis, synthesis, or application of the presented material.
Team-based learning	Education strategy where students are placed on small teams to collaboratively solve problems. This approach may involve pre-class preparation and individual readiness assessments prior to group work.

Besides the relationship between curriculum models and their relative influence on shelf scores, it is also worth investigating what other external factors may influence a student’s success. One area of research has been the timing of the shelf exam, such as the length of the clerkship or where the clerkship falls in a student’s respective schedule. Interestingly, the results for the prior have varied greatly. On one hand, there is evidence to suggest that a shorter time to shelf negatively impacts scores with students at Mayo who completed 3–4 weeks of psychiatry scoring significantly lower than those completing 6 weeks [38]. Another study from Columbia found that students on the longitudinal surgical curriculum outperformed those on the standard 12-week program [39]. On the other hand, when the University of Michigan shortened all clerkships by 25%, it did not result in significant changes on any of the NBME shelf exams [40]. Additionally, due to COVID, an internal medicine clerkship that was shortened to 5 weeks at one institution as opposed to the normal 8 weeks showed no statistical difference in scores [41] and in support of this, there was a separate multi-institutional analysis that looked at the length of internal medicine clerkships and exam performance and found no significant association [9]. Therefore, it remains unclear what the optimal length is for a clerkship in terms of eventual success on the shelf exam.

What does appear clear in the research is the positive association between the number of prior shelf exams a student has taken and a higher score. Numerous studies have observed this correlation spanning across multiple specialties. The total number of core clerkship students completed before the neurology shelf exam was associated with higher scores [42], and similar results have been reported in psychiatry [35,43]. The multi-institutional study of internal medicine clerkships referenced above also found that examinees who took the shelf later in the year scored significantly higher [9]. Additionally, there have been numerous single-institution studies demonstrating that students who have taken internal medicine do better on their subsequent surgical shelf examination [21,44,45]. To our knowledge, there are no studies that show a negative correlation between the number of clerkships completed and exam scores.

Although students’ specific schedules are outside the control of clerkship directors, understanding that students who take a clerkship earlier in the year are at a disadvantage is still useful knowledge. Clerkship directors can counsel students on this when they are creating their schedules, so they may consider placing specialties they are highly interested in later in the year and also provide extra academic support to students during clerkships earlier in the year.

### Addressing criticism

Several findings discussed in this review may be unexpected to medical educators despite being supported by the existing literature, and we anticipate that some of these concerns may invite criticism or alternative interpretations. As with any body of research, the potential for bias must also be considered. For example, the observation that external factors such as examination timing can influence shelf exam performance may raise concern regarding measurement bias. If shelf examinations are intended to provide an objective assessment of student knowledge, it is reasonable to question why factors unrelated to content mastery should affect scores. While a comprehensive discussion of this issue is beyond the scope of this review, one possible explanation is that medical knowledge is cumulative and highly interconnected. Improved performance among students who have completed a greater number of clerkships may reflect the intended function of shelf exams: assessing a learner’s ability to integrate and apply knowledge across increasingly complex clinical contexts. However, other explanations are also plausible and demonstrate the ongoing need for research in this area to ensure fair assessments for all students.

We are additionally aware that our paper may be criticized for encouraging educators to ‘teach to the test.’ This concern is weakened, however, if we believe that the test is capturing the knowledge it ought to. While questions can always be raised about the quality of medical school examinations, medical educators continuously including these exams in their evaluations of students signals their approval of the shelf exams as evaluative measures of a student’s knowledge base. Furthermore, as we outline at the start of the paper, so long as Step 2 continues to be a strong determinant of a student’s ability to successfully match into residency, medical educators have a duty to properly prepare their students, which appears to include utilizing the shelf exams. Despite our defence, we acknowledge that examinations are only one part of the puzzle when it comes to crafting a physician, and our paper should not be taken as signalling disapproval of concurrently focusing on the softer skills of medicine. Nor should our paper be taken as dissuading medical educators from emphasizing clinical reasoning and problem-solving in their curriculum. Rather, we aim to raise awareness on the current body of evidence pertaining to shelf exam preparation and subsequent Step 2 success while also urging for continued research in the field at large.

## Conclusion

There is strong evidence to suggest that foundational knowledge of disease pathology is essential for success on shelf exams and should be top of mind for clerkship directors while preparing their students not only for the final exam on a specific rotation but also for their eventual national licensing exams. There is also strong evidence that the number of core clerkships students have completed will ultimately enhance their performance, and moderate evidence that lectures focused on active learning, while not improving performance, are well received.

These findings are clearer cut when compared to research on how clinical reasoning and exposures impact future scores. However, shelf exams are not the only priority of clerkship directors, who also aim to form medical students into clinically competent and compassionate future physicians. Nonetheless, the objective nature of the shelf exam makes it a valuable tool for navigating a post-scored Step 1 world, as shelf exams both offer students a sense of how they may perform on Step 2 and contribute to achieving honours. Although to this latter point, while the shelf exam itself is an objective measure, how it is implemented into the final grade varies greatly at different institutions, with some institutions not incorporating honours into their grading at all [46].

This literature review highlights the need for continued research on how clerkship directors may make changes that ultimately balance the goal of understanding basic pathophysiology and applying this knowledge to succeed on exams, with the goal of crafting future physicians with broad and practical clinical knowledge.

## Data Availability

There is no primary data associated with this research.
